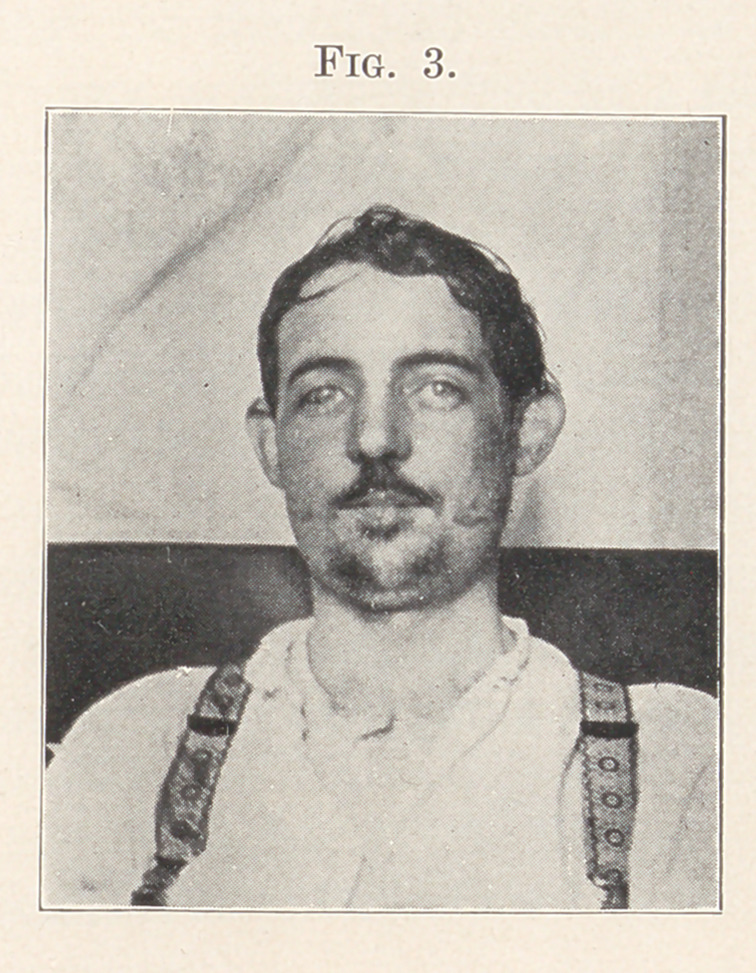# Multiple Fracture of the Lower Jaw Complicated by Double Fracture of the Upper Jaw

**Published:** 1904-11

**Authors:** Thomas L. Gilmer

**Affiliations:** Chicago


### MULTIPLE FRACTURE OF THE LOWER JAW COMPLICATED BY DOUBLE FRACTURE OF THE UPPER JAW.[1]

BY THOMAS L. GILMER, M.D., D.D.S., CHICAGO.

THE following case, with treatment, is considered of sufficient interest to present for your consideration:

From St. Luke's Hospital record of May, 1902, is taken the following:

---

[1] Read at the annual session of the American Medical Association, Section on Stomatology, Atlantic City, June 7 to 10, 1904.

"*Patient.*—G. C. was brought to accident ward about one A.M., May 12, 1902, in police ambulance, presenting the following lesions:

"1. Fracture of lower jaw multiple: (*a*) just to left of symphysis between two left incisors, compound in mouth; (*b*) on right side between bicuspid and first molar; (*c*) at angle of jaw right side, simple; whole lower jaw was flattened antero-posteriorly and dropped somewhat toward sternum.

"2. Fracture of upper jaw: (*a*) palate and alveolar processes broken from attachments and freely movable; (*b*) both alveolar processes broken loose from palate; (*c*) hard palate seemed fractured—antero-posteriorly throughout nearly the whole length—near the middle line. (Later examination did not positively confirm this.)

"3. Loss of teeth. Upper jaw: (*a*) four incisors; (*b*) both canines. Lower jaw: (*a*) left central incisor.

"4. Anterior portion of upper alveolar process detached from bone above and space so formed communicated freely with right nostril and probably with right antrum of Highmore.

"5. Extensive laceration of lower lip.

"6. Incised wound of chin.

"7. Hemorrhage from right ear not manifested until twelve hours after accident.

"8. Hemorrhage into soft parts about both eyes and into conjunctivæ.

"9. Hemorrhage subcutaneous about both ears.

"*Cause of Injury and Emergency Treatment.*—Fall from second-story window on a stone pavement. History of intoxication obtained from room-mate and confirmed by breath. Was in a heavy stupor, not unconscious, rather restless, and was continually spitting blood. Lower lip and wound of chin sutured with silk-worm gut, about three sutures in each, in accident ward. Hemorrhage from nose and mouth was free and showed no tendency to stop. Both nostrils packed with iodoform gauze about three A.M. Hemorrhage only partly checked, and about five A.M. space leading up to nose, above the fractured alveolar process, was tightly packed with iodoform gauze. Hemorrhage then ceased after sufficient loss of blood to be appreciated constitutionally. Patient became more wideawake towards morning and has remained conscious and rational. Was seen by Dr. Gilmer about two P.M., who wired, provisionally, the two teeth at sides of fracture near symphysis. Wet cold compresses were applied over face, and mouth and nose were thoroughly irrigated t. i. d. with saturated boric solution, two ounces; oil cassia, one drop. Ear cleaned, filled with boric powder, and drained.

"Rectal temperature, 100.4; pulse, 86; respiration, 20."

*Examination.*—After a careful examination of the patient I found that the nature of the fracture and injuries of the jaws were more extensive than history of the internes indicated. I found the man in a semicomatose condition, blood oozing from the right ear, and also a conjunctival ecchymosis. His condition was so serious that I concluded absolute quiet more important for a few days than the setting of the bones of the jaws and face; indeed, I was quite positive that he could not recover. All of the injured parts were maintained in as nearly an aseptic condition as possible, the bowels kept open, and he was kept quiet. On the fifth day he had improved to such an extent that I felt it was safe to proceed with the treatment of the fractures.

A corrected diagnosis showed that there were five fractures of the lower jaw. On the left side, one at the angle, another on the line of the first bicuspid. On the right side there was a break at the neck of the condyle, one at the angle, and one at the cuspid tooth. The upper jaw was broken in half, through the median line, and the two halves were broken from their attachment above. All of the incisors and both cuspids on the upper jaw were knocked out and lost; the other teeth on this jaw were in place. On the lower jaw, strangely, only one tooth, an incisor, was missing. Those on a line with the fracture in the body of the bone were loosened.

*Treatment.*—In such a case no one method of treatment is applicable, therefore I decided on a combination of wiring and splinting, hoping by this means to at least partially restore the contour of the face and get a reasonably good occlusion of the teeth. Looking to this end, assisted by Dr. Arthur D. Black, an impression was made of the upper jaw and teeth in very soft modelling composition, the two lateral halves of the jaw having been temporarily restored to their normal position. On a cast from this impression a modified Kingsley splint was formed of vulcanite, square brass tubes being vulcanized in the splint on each side to receive the side arms. On the lower part of this splint wire staples were secured to receive the wires which were to be attached to the lower teeth (Fig. 1).

Holes were now drilled through the bone on either side of the anterior fracture on the lower jaw and the fragments caused by this break securely wired to each other by heavy silver wire. This gave stability to a considerable portion of the body of the bone. German silver wires were now placed around the necks of the firmly set teeth on the lower jaw and secured by twisting. The splint was adjusted to the upper teeth and the two halves of the upper jaw were drawn forward and pressed upward in their normal relation with the bones above it and secured in place by laces extending from the side arms, to which they were attached to eyelets in a skull cap (Fig. 2). The wires on the lower teeth were now secured to the staples on the lower part of the splint, the lower jaw being drawn forward by this attachment to its normal position and the teeth held in apposition with the splint, the lower surface of which was shaped to correspond with what I believe to represent the normal occlusion.

*Results.*—The result was far more satisfactory than could reasonably have been anticipated, and is fairly well shown in Fig. 3, made from a photograph taken immediately after the removal of the appliances. The occlusion of the teeth was so nearly correct that a trifle grinding made it approximately perfect. To prevent the laces slipping on the metal arms extending out from the mouth, pieces of adhesive plaster were attached to them. Through these holes were punched to receive the laces (Fig. 2).

The progress of the case was uneventful, the temperature never rising above 100.6. Primary union was secured in each fracture, and the patient was discharged one month and three days after the application of the splint and the wiring of the bone and teeth.

### DISCUSSION.

*Dr. G. V. I. Brown,* Milwaukee.—I suppose it is only those who have been through the trials of excessive fractures and who are familiar with the conditions under which such cases come to the stomatologist and oral surgeon for treatment who can appreciate the very great difficulties that Dr. Gilmer has so successfully overcome in this case. Any one of the five fractures that was reported in this one jaw would have been sufficient difficulty in itself, but to take a multiple fracture with surrounding tissue having its in-

Fig. 1.

Fig. 2.

Fig. 3.

tegrity almost entirely destroyed by the traumatism makes the appearance of the patient as shown in the final result something almost beyond expectation. Commonly, the teaching with regard to the treatment of fractures is that it is necessary to apply the splint almost immediately. I note that Dr. Gilmer found it necessary to allow several days to elapse, and I think that that is the part of wisdom. Not only was it so in this case, but I believe it to be true in many other fractures of less degree. I have found it extremely useful to construct a temporary splint of modelling compound. I always carry this modelling compound in my surgical bag, and for use in an emergency it is extremely valuable. It can be softened in hot water and placed before there has been opportunity for swelling, soreness, or other complication to arise. When these conditions have subsided and the patient is in condition to control with comparative comfort, a better splint can be constructed, but even without the construction of another splint, modelling compound can be made to serve the purpose.

## Figures and Tables

**Fig. 1. f1:**
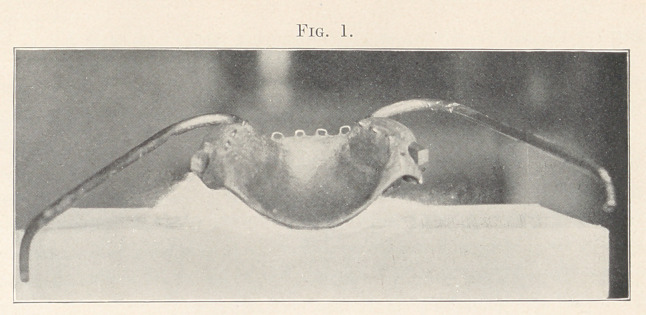


**Fig. 2. f2:**
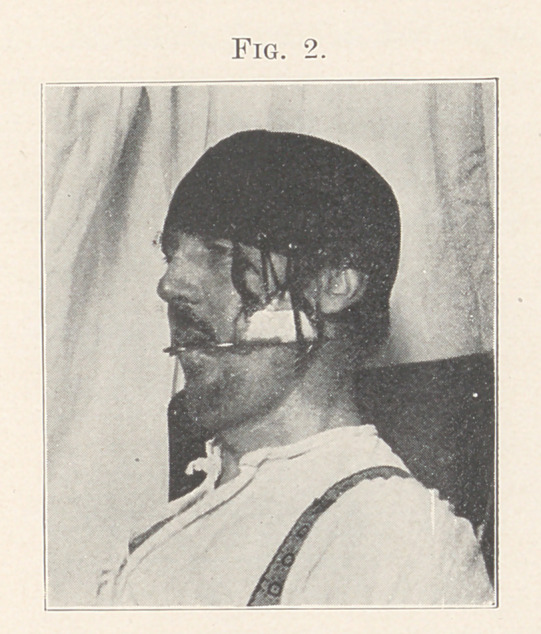


**Fig. 3. f3:**